# Polymicrobial Biofilm Interaction Between *Histophilus somni* and *Pasteurella multocida*

**DOI:** 10.3389/fmicb.2020.01561

**Published:** 2020-07-10

**Authors:** Briana Petruzzi, Allan Dickerman, Kevin Lahmers, William K. Scarratt, Thomas J. Inzana

**Affiliations:** ^1^Department of Biomedical Sciences and Pathobiology, Virginia-Maryland College of Veterinary Medicine, Virginia Tech, Blacksburg, VA, United States; ^2^Biocomplexity Institute and Initiative, University of Virginia, Virginia Tech, Charlottesville, VA, United States; ^3^Department of Large Animal Clinical Sciences, Virginia-Maryland College of Veterinary Medicine, Blacksburg, VA, United States; ^4^Department of Veterinary Biomedical Sciences, College of Veterinary Medicine, Long Island University, Brookville, NY, United States

**Keywords:** biofilm, polymicrobial, bovine respiratory disease, *Histophilus somni*, *Pasteurella multocida*

## Abstract

*Histophilus somni* and *Pasteurella multocida* are two of multiple agents responsible for bovine respiratory disease (BRD) in cattle. Following respiratory infection of calves with *H. somni*, *P. multocida* may also be isolated from the lower respiratory tract. Because *H. somni* may form a biofilm during BRD, we sought to determine if *P. multocida* can co-exist with *H. somni* in a polymicrobial biofilm *in vitro* and *in vivo*. Interactions between the two species in the biofilm were characterized and quantified by fluorescence *in situ* hybridization (FISH). The biofilm matrix of each species was examined using fluorescently tagged lectins (FTL) specific for the exopolysaccharide (EPS) using confocal laser scanning microscopy. Bacterial interactions were determined by auto-aggregation and biofilm morphology. *Pasteurella multocida* and *H. somni* were evenly distributed in the *in vitro* biofilm, and both species contributed to the polymicrobial biofilm matrix. The average biomass and biofilm thickness, and the total carbohydrate and protein content of the biofilm, were greatest when both species were present. Polymicrobial bacterial suspensions auto-aggregated faster than single species suspensions, suggesting physical interactions between the two species. Almost 300 *P. multocida* genes were significantly differentially regulated when the bacteria were in a polymicrobial biofilm compared to a mono-species biofilm, as determined by RNA-sequencing. As expected, host genes associated with inflammation and immune response were significantly upregulated at the infection site following *H. somni* challenge. Encapsulated *P. multocida* isolates not capable of forming a substantial biofilm enhanced an *in vitro* polymicrobial biofilm with *H. somni*, indicating they contributed to the polymicrobial biofilm matrix. Indirect evidence indicated that encapsulated *P. multocida* also contributed to a polymicrobial biofilm *in vivo*. Only the EPS of *H. somni* could be detected by FTL staining of bovine tissues following challenge with *H. somni*. However, both species were isolated and an immune response to the biofilm matrix of both species was greater than the response to planktonic cells, suggesting encapsulated *P. multocida* may take advantage of the *H. somni* biofilm to persist in the host during chronic BRD. These results may have important implications for the management and prevention of BRD.

## Introduction

Bovine respiratory disease (BRD) complex causes significant economic losses to the beef industry through increased treatment costs, reduced carcass value due to treatment and prevention measures, morbidity, and mortality (Griffin, 1997; Stovall et al., 2000). BRD is a collective term describing respiratory infections from causative agents including the predominant bacterial species *Histophilus somni*, *Pasteurella multocida*, *Mannheimia haemolytica*, and *Mycoplasma bovis*, as well as several viruses (Griffin et al., 2010). It is common to detect more than one causative agent during an outbreak (Gershwin et al., 2005; Booker et al., 2008; Agnes et al., 2013), suggesting BRD is often polymicrobial.

A definitive diagnosis of BRD can only be made after post-mortem examination, resulting in broad, poorly defined diagnostic criteria (Taylor et al., 2010). Signs of disease can go undetected, and include a lack of appetite, nasal discharge, coughing, rapid breathing, fever, and diarrhea. Polymicrobial infections can display a more complex pathology than single species infections (Sibley et al., 2008; Burmølle et al., 2014; Murray et al., 2014), and are more resistant to antibiotics (Perez et al., 2014). Cattle are predisposed to disease after experiencing stress, including situations such as weaning, excessive handling, a change in diet, and transportation or exposure to new locations and/or herds (Snowder et al., 2006; Holman et al., 2017). Genetic factors may also predispose cattle to disease (Snowder et al., 2006). It is generally accepted that transmission occurs via contaminated aerosols in locations with limited ventilation such as during transportation, in auction houses or crowded barns, or after a change in climate or location.

All of the primary bacterial agents responsible for BRD have been demonstrated to produce biofilms (Sandal et al., 2007; Simmons and Dybvig, 2009; Boukahil and Czuprynski, 2015; Petruzzi et al., 2017), which may be associated with their pathogenic role during BRD. *H. somni* biofilm formation has been directly associated with BRD (Sandal et al., 2009). Bacterial populations within a biofilm are pleural in respect to their phenotype and genotype (Ehrlich et al., 2005), and therefore the study of bacteria in a monophasic system (e.g., a planktonic log phase culture) may be highly irrelevant to their status *in vivo*. Prior experimental bovine respiratory challenge showed that during chronic infection *H. somni* exists as a biofilm in the cardiopulmonary tissue of calves raised from birth and in isolation (Sandal et al., 2009). However, *P. multocida* was also isolated from these biofilm samples, as well as from other *H. somni* respiratory challenge experiments (Gogolewski et al., 1987a, a; Elswaifi et al., 2012; Murray et al., 2017). BRD research over the past 4 decades has aided in the reduction of disease incidence, provided a better understanding of disease pathogenesis and the immune response, aided in the detection of new infectious agents, and has promoted the implementation of preventative techniques by veterinarians. However, despite such progress, BRD is still the largest cause of morbidity and mortality in the beef industry and current vaccines offer little relief, indicating the need for further research (Griffin, 1997; Fulton, 2009; Taylor et al., 2010).

The work described in this report aims to characterize the interactive polymicrobial relationship between the BRD pathogens *H. somni* and *P. multocida* during biofilm formation. Confocal laser scanning microscopy (CLSM) of fluorescently labeled components enabled each species to be observed simultaneously within the biofilm matrix, providing a detailed understanding of their polymicrobial relationship. Additionally, data obtained from an experimental infection of calves supported the co-habitation of *H. somni* and *P. multocida* in polymicrobial biofilms *in vivo*. The information presented here further supports the role of biofilm as an important phase of BRD, provides evidence that BRD can be a complex polymicrobial biofilm disease, and supports the need for treatment and preventative measures targeting biofilm formation.

## Materials and Methods

### Bacterial Growth

*Histophilus somni* strain 2336, *Pasteurella multocida* strain C0513, and capsule-deficient variant C0513-P5 (isolated after 5 serial passages of a single colony of C0513 to obtain a rough colony variant) were used (Petruzzi et al., 2017). *H. somni* was grown in Brain Heart Infusion (BHI) broth (BD Scientific; Irvine, CA, United States) containing 0.5% yeast extract (BD Scientific; Irvine, CA, United States), 0.1% Trizma base, 0.01% thiamine monophosphate (TMP) (Sigma-Aldrich, St. Louis, MO, United States), and 1% bovine serum at 37°C with rapid shaking until growth reached 10^9^ colony forming units (CFU)/ml (determined by spectrophotometric reading and confirmed by viable plate count). *P. multocida* strains C0513 and C0513-P5 were grown in BHI broth alone, incubated and shaken as above to the same density. Biofilms were grown in 50-mL conical tubes using the same medium at 37°C with rotation at 50 rpm or less. *H. somni* biofilms were grown for approximately 3 days (Sandal et al., 2011) before the addition of *P. multocida* to obtain polymicrobial biofilm growth. After the addition of *P. multocida*, biofilm incubations were continued for an additional 2 days to enable the *P. multocida* biofilm to reach maturity (Petruzzi et al., 2017). Single species biofilms were grown for 5 days for *H. somni*, and 2 days for *P. multocida* due to the difference in growth rate of the two species.

### Fluorescence *in situ* Hybridization

Fluorescence *in situ* hybridization (F.I.S.H.) was performed using the following 16S rRNA probes: ′/5FluorT/TT AAG AGA TTA ATT GAT TGA′ to detect *H. somni*, and ′/5Cy5/CT ATT TAA CAA CAT CCC TTC′ to detect *P. multocida*. DNA-specific probes were purchased from Integrated DNA Technologies Inc. (Coralville, IA, United States), and confirmed to be species-specific using a BLAST search and through experimental hybridization with homologous and heterologous bacterial species. F.I.S.H. was carried out as described (Thurnheer et al., 2004), with the following modifications. The hybridization buffer determined to optimize attachment of both probes contained 25% formamide. Biofilms were grown on glass coverslips and fixed with 4% paraformaldehyde for at least 60 min. Biofilms were incubated with hybridization buffer for at least 15 min at 37°C. After pre-incubation, 0.2 μg of each probe was added to the fixed biofilm and incubated overnight at 37°C. Biofilms were then washed 1× with wash buffer, then rinsed thoroughly with water. Glass coverslips and slides were embedded biofilm-side down with 20 μl of polyvinyl alcohol (Mowiol^®^ 4–88; Millipore Sigma-Aldrich, St. Louis, MO, United States) onto glass slides, and stored in the dark. CLSM was performed on a Zeiss 880 confocal laser scanning microscope (Zeiss; Oberkochen, Germany) at 40× magnification using Cy5 (deep red, 660–710 nm) and fluorescein (green, 500–540 nm) filters.

### Fluorescence Lectin Staining of the Biofilm Exopolysaccharide

Fluorescein isothiocyanate (FITC)-conjugated *Griffonia simplicifolia* lectin (GS-II) (EY laboratories; San Mateo, CA, United States) was used to detect glycogen EPS (Hennigar et al., 1986, 1987) produced by *P. multocida* (Petruzzi et al., 2017). Tetramethylrhodamine (TRITC)-conjugated Moringa M Lectin (MNA-M) (EY laboratories) was used to detect the *H. somni* galactomannan EPS (Sandal et al., 2009). Lectins were suspended in 10 mM phosphate buffer, pH 7.5, at a concentration of 10 μg/ml. Fifty microliters of lectin solution was applied to the biofilms and incubated 30–60 min in the dark at room temperature, then washed with phosphate buffer four times. Coverslips were fixed onto slides with 20 μl of polyvinyl alcohol and stored in the dark for at least 6 h before imaging. CSLM was performed on a Zeiss LSM 880 CSLM (Zeiss) at 40× magnification using TRITC (red, 532 nm) and fluorescein (green, 500–540 nm) filters.

### Polymerase Chain Reaction

The primers specific to the *P. multocida* and *H. somni* 16S rRNA gene that were used in this study are described elsewhere (Angen et al., 1998; Mbuthia et al., 2001). Isolated colonies were suspended in sterile water and boiled for 10 min. Unbroken cells were removed by centrifugation at 12000 rpm for 5 min, and the supernatant used as a DNA template for PCR amplification. DNA was extracted from sections of bovine lung tissue using the Blood and Tissue DNeasy Kit (Qiagen) according to manufacturer’s instructions. PCR was performed on extracted DNA material using OneTaq Kit (NEB), according to manufacturer’s instructions.

### Enzyme-Linked Immunosorbent Assay

Enzyme-linked immunosorbent assays (ELISA) were performed to determine the antibody response to *H. somni* whole cells, *P. multocida* whole cells, or crude single species biofilm material. Biofilms were grown for 5 days, most of the fluid aspirated, the matrix material suspended in distilled water and lyophilized, and each sample resuspended in sterile saline to 1 mg/ml. Twenty micrograms of this biofilm material per well were used to coat the surface of Immulon 4HBX plates (Thermo Fisher Scientific). Bacteria were grown to 10^9^ CFU/ml (mid-log phase), then resuspended to the same concentration in carbonate coating buffer, pH 9.6 (15 mM Na_2_CO_3_, 35 mM NaHCO_3_, 3 mM NaN_3_). Wells were coated with 10^8^ CFU of whole cells. Phosphate buffered saline (PBS) supplemented with 0.05% Tween 20 (Sigma) and 2% non-fat dry milk (Kroger) was used as a blocking agent. All washes between each step (5× each) were carried out with PBS supplemented with 0.05% Tween-20. The primary antibody used was calf serum that was separated from whole blood at the time of euthanasia. The secondary antibody used was rabbit IgG to bovine heavy & light chain conjugated to horseradish peroxidase (Jackson laboratory; Bar Harbor, ME, United States). TMB substrate (Pierce Biotechnology; Rockford, IL, United States) was used for color development, per the manufacturer’s instructions. Control wells included all components except the initial biofilm antigen.

### Biofilm Protein and Carbohydrate Concentrations

The protein and carbohydrate content of the biofilms was determined using the BCA assay (Thermo Fisher Scientific) and the Anthrone assay (Yemm and Willis, 1954; Laurentin and Edwards, 2003), respectively. Equal concentrations of biofilm material were suspended in 1 ml of PBS, and vortexed rapidly for several seconds to break up the biofilm matrix prior to assay.

### Auto-Aggregation of Single Species and Polymicrobial Suspensions

Auto-aggregation was performed as previously described (Sorroche et al., 2012) with modifications. Bacterial cells from *P. multocida* C0513, C0513-P5, *H. somni* 2336, or in combination were suspended in sterile PBS with or without supplementation with 100 μg/ml of *H. somni* crude biofilm material. OD_562_ measurements were taken prior to the start of the experiment, after 1 h, and after 24 h. The initial OD_562_ was between 0.8 and 1.0. The auto-aggregation percentage at each time period was calculated from [1 - (OD_final_/OD_initial_)] × 100.

### Determination of Polymicrobial Biofilm Formation *in vivo*

Two, eight-week old, male Holstein calves (#65 and 66) were challenged with 10^9^ CFU of *H. somni* strain H.S.25 in 10 ml sterile PBS containing 0.15 mM CaCl_2_ and 0.5 mM MgCl_2_ (PCM) intratracheally using a syringe (6–12 cc) and a Tom Cat/Small Animal Catheter (3.5 Fr, 5.5 inches long). Calves were monitored daily for signs of respiratory infection. Twenty-two days after challenge, calves were humanely euthanized, and the lungs were harvested for further analysis. Bacterial load within the pulmonary tissue was determined by manually breaking the lung tissue in sterile PCM buffer. The resulting digest was serially diluted. Dilutions were spread onto Columbia blood agar and incubated for 72 h at 37°C in 6% CO_2_. The presence of *H. somni* and *P. multocida* was determined by colony morphology upon isolation and subsequent PCR of isolated colonies and lung tissue. Tissue sections were embedded in paraffin and cut into 5 μm sections on glass slides. The slides were deparaffinated and stained by F.I.S.H. and fluorescently tagged lectins as described above. Histology was carried out on sections of paraffin-fixed tissue that were stained with hematoxylin and eosin.

### RNA-Sequencing (RNA-Seq)

RNA was isolated from bacterial biofilms in triplicate on 3 separate occasions using Qiagen RNA Protect bacterial reagent, Qiagen QiaShredder, and Qiagen RNeasy kits (Qiagen; Hilden, Germany) according to manufacturer’s instructions for prokaryotic RNA. The isolated RNA was immediately transcribed into cDNA using the Quanta qScript kit (Quanta Biosciences; Gaithersburg, MD, United States) following the manufacturer’s instructions.

Lung samples collected at the time of euthanasia were stored in RNA*later* RNA storage reagent (Qiagen; Hilden, Germany) for extraction of host RNA. Lung samples collected at the time of euthanasia were also stored in both liquid nitrogen and 10% buffered formalin for further analysis. Twenty 50-mg sections of lung were homogenized in 900 μl TriZol (Thermo Fisher Scientific; Waltham, MA, United States), and stored at room temperature for several minutes. Chloroform (180 μl) was then added to the homogenate, and vortexed vigorously. After 3 min at room temperature, the homogenate solutions were centrifuged at 12,000 × *g* for 15 min at 4°C. The aqueous phase was transferred to a clean tube containing an equal volume of 70% ethanol, and the RNA extracted with the Qiagen RNeasy kit (Qiagen; Hilden, Germany). The purity of the RNA was determined by the A_260/280_ and A_260/230_ ratios using nanodrop (NanoDrop; Wilmington, DE, United States). Fifty ng of pure RNA was transcribed into cDNA using the Quanta Biosciences qScript^TM^ XLT cDNA Supermix kit (Quanta Biosciences; Gaithersburg, MD, United States) according to the manufacturer’s instructions.

Quantitative real-time polymerase chain reaction (qRT-PCR) of the 16S rRNA gene was used as a control for RNA-Seq. qRT-PCR was performed using the Quanta Biosciences PerfeCta^®^ SYBR^®^ Green FastMix, ROX^TM^ (Quanta Biosciences; Gaithersburg, MD, United States) according to the manufacturer’s instructions on an Applied Biosciences 7300 Real-Time PCR system (Applied Biosciences; Foster City, CA, United States). Two oligonucleotide probes specific for the 16S rRNA gene of *H. somni* (5′-TTCGGGCACCAAGT(A/G) TTCA-3′) (Angen et al., 1998) and *P. multocida* (5′-ACGAGACTCTAGACTCCC-3′) (Mbuthia et al., 2001), respectively, were obtained from MWG-BIOTECH AG, Ebensburg, Germany and were labeled with the isothiocyanate derivative Cy3. Quantitative RT-PCR was performed in 20 μl reactions using 50 ng cDNA template and 300 mM of each primer. Genetic data were annotated using the Database for Annotation, Visualization, and Integrated Discovery (DAVID) v6.8^1^.

The original RNA-Seq data presented in this study are publicly available. These data can be found at the following NCBI SRA site^2^.

### Statistical Analysis

Analysis of polymicrobial biofilms was performed on *z*-stack images obtained by CLSM. *z*-stacks were analyzed using COMSTAT coded files (Heydorn et al., 2000) through MATLAB software (MathWorks; Natick, MA, United States). All measured experiments were performed in triplicate in three different experiments on different days (9 datum points total). Statistical analysis was performed using GraphPad Prism software version 6.01 (GraphPad; La Jolla, CA, United States). Statistical significance was considered if *p*-values were ≤0.05.

## Results

### Fluorescence *in situ* Hybridization (F.I.S.H.) of Polymicrobial Biofilms Using DNA-Specific Probes

**FIGURE 1:**
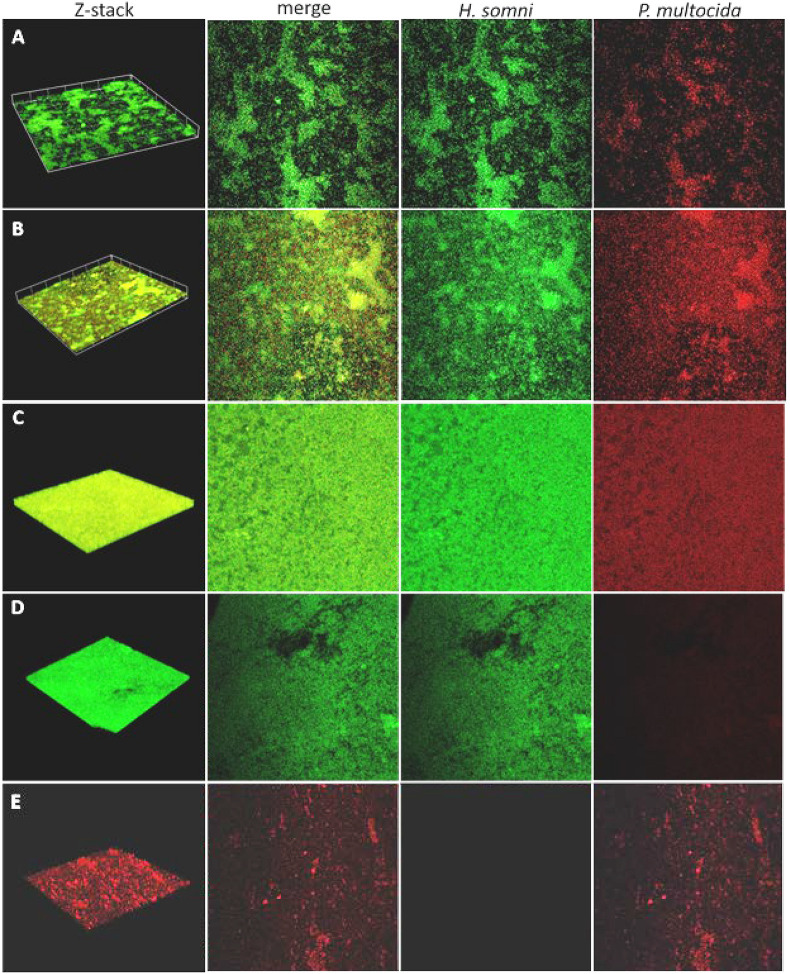
Fluorescence *in situ* hybridization (FISH) of polymicrobial and single species biofilms. Biofilms were imaged over a 48-h period by CLSM after FISH using probes labeled with fluorescein for *H. somni* or Cy5 for *P. multocida*. **(A–C)**, 6-h, 12-h, and 48-h time points, respectively, for biofilms containing *H. somni* strain 2336 and *P. multocida* strain C0513-P5. Each column shows the 3-D z-stack, a merged view of images with each probe, the probe for only *H. somni* strain 2336, and the probe for only *P. multocida* strain C0513-P5. **(D)** Single species biofilm of *H. somni* strain 2336 after 48 h showing in each column the 3-D z-stack, the merged view of images with each probe, the probe for only *H. somni* strain 2336, and the probe for only *P. multocida* strain C0513-P5. **(E)** A single species biofilm of *P. multocida* strain C0513 after 48 h showing in each column the 3D z-stack, the merged view of images with each probe, the probe for only *H. somni* strain 2336, and the probe for only *P. multocida* strain C0513-P5.

**FIGURE 2:**
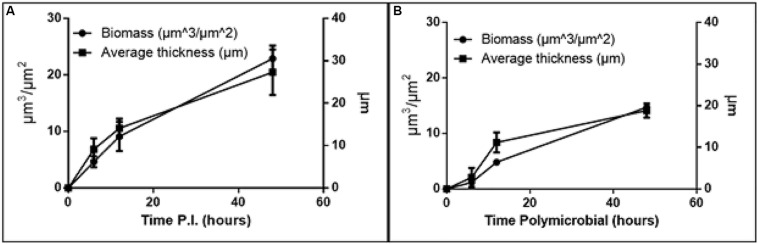
Increase in biomass and thickness within the polymicrobial biofilm. **(A)** *H. somni* strain 2336 biomass and thickness within the polymicrobial biofilm. **(B)** *P. multocida* strain C0513-P5 biomass and thickness within the polymicrobial biofilm. All experiments were performed in triplicate in three different experiments (9 datum points total).

Fluorescent DNA probes specific for the 16S rRNA region of *H. somni* or *P. multocida* DNA were used to determine the spatial arrangement of *P. multocida* within an established *H. somni* biofilm. After 48 h, *P. multocida* integrated evenly throughout the *H. somni* biofilm (Figures 1A–C). Large micro-colonies of *P. multocida* were not visible, which has been reported for other polymicrobial bacterial biofilms (Zainal-Abidin et al., 2012; Zhu et al., 2013). The *H. somni* biofilm continued to mature after the addition of *P. multocida* over the monitored 48-h period, indicated by increased green fluorescence between time points (Table 1, Figure 2A). COMSTAT analysis was used to determine the three-dimensional spatial occupation (biomass) of the DNA from each species, as well as the two dimensional thickness of the DNA occupied by each species (Table 1). Thickness was used as a determination of the depth to which *P. multocida* had integrated into the established biofilm matrix.

**TABLE 1:** COMSTAT analysis of fluorescence *in situ* hybridization of polymicrobial and single species biofilms.^a^

Time polymicrobial	Biomass (μm^3^/μm^2^)	Average thickness (μm)	Roughness coefficient (0–2)	Surface to biovolume ratio (μm^2^/μm^3^)
***H. somni***				
6 h	4.63 ± 0.96	9.13 ± 2.6	0.29 ± 0.07	3.5 ± 0.34
12 h	9.11 ± 2.57	14.1 ± 2.22	0.11 ± 0.05	2.460.32
48 h	22.89 ± 2.3	27.32 ± 5.36	0.04 ± 0.01	1.26 ± 0.48
***P. multocida* C0513**				
6 h	1.18 ± 0.78	2.77 ± 2.29	1.3 ± 0.41	5.57 ± 2.05
12 h	4.84 ± 0.14	11.17 ± 2.40	0.16 ± 0.05	3.39 ± 0.32
48 h	14.47 ± 0.48	18.85 ± 1.70	0.08 ± 0.01	1.58 ± 0.01
**Single species at 48 h**				
*H. somni*	11.990.75	17.79 ± 2.44	0.09 ± 0.02	1.64 ± 0.1
*P. multocida* CO513-P5	9.61 ± 0.5	16.05 ± 0.90	0.07 ± 0.002	5.22 ± 0.09

*^*a*^Data was collected over 48 h of biofilm growth.*

The *H. somni* biomass increased from 4 ± 1 μm^3^/μm^2^ at 6 h to 22 ± 2 μm^3^/μm^2^ at 48 h (Table 1, Figure 2A). The average thickness of the *H. somni* biomass also increased over this 48 h period. The roughness coefficient is an arbitrary value used to determine the topography of the biofilm surface, which remained relatively constant throughout the experiment (Table 1). Therefore, the addition of *P. multocida* did not significantly alter biofilm architecture. The surface to bio-volume ratio decreased over the 48 h period, indicating biofilm growth increased (Table 1). The values obtained using the *H. somni* DNA probe were used to determine the total thickness of the biofilm, while the total biomass was determined by the addition of the *H. somni* and *P. multocida* biomass values.

Six hours after its addition to the established *H. somni* biofilm, *P. multocida* attached to the *H. somni* biofilm, as an apparent monolayer (Figure 1A). This is indicated by a *P. multocida* thickness measuring 2.77 ± 2.29 μm. The *P. multocida* biomass was determined to be 1.18 ± 0.78 μm^3^/μm^2^ at 6 h. However, at the 48 h polymicrobial time point, *P. multocida* had expanded to a thickness of 18.85 ± 1.7 μm and a biomass of 14.76 ± 0.48 μm^3^/μm^2^ (Table 1, Figures 1C, 2B), indicating that *P. multocida* had incorporated into 69% of the total *H. somni* biofilm matrix and contributed to 39% of the total biomass. Over the 48 h period, the roughness coefficient of the *P. multocida* biofilm contribution decreased to values similar to the *H. somni* roughness coefficient (Table 1), indicating that *P. multocida* conformed to the *H. somni* biofilm structure. Despite their apparent cooperation, the polymicrobial biofilm did not appear to provide a growth advantage to either individual species. The biomass and thickness values for the polymicrobial biofilm (indicated by the values provided for *H. somni* within the polymicrobial biofilm) were not significantly different than the addition of the values for each single species biofilm (Table 1, Figures 1D,E).

### Fluorescent-Tagged Lectin Staining of Biofilm EPS

**FIGURE 3:**
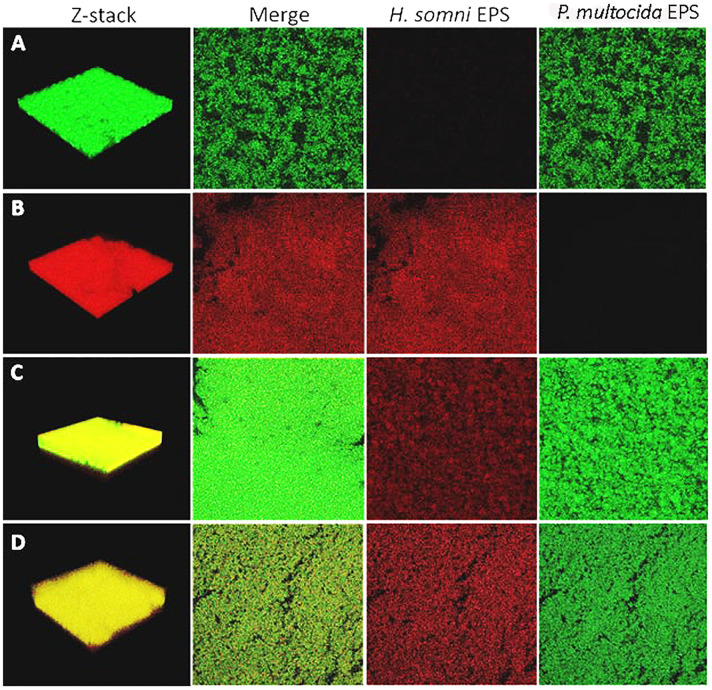
Fluorescently tagged lectin staining of in vitro biofilm EPS. *Moringa* M labeled with TRITC was used as the specific lectin for the galactomannan EPS from *H. somni*, and *Griffonia simplicifolia* labeled with FITC was used as the specific lectin for the glycogen EPS of *P. multocida.* **(A)** *P. multocida* strain C0513-P5 single species biofilm Z-stack, the merged view of images with each lectin, *H. somni* strain 2336 EPS, *P. multocida* strain C0513-P5 EPS. **(B)** *H. somni* strain 2336 single species biofilm Z-stack, the merged view of images with each lectin, *H. somni* strain 2336 EPS, *P. multocida* strain C0513-P5 EPS. **(C)** Polymicrobial biofilm with *P. multocida* C0513-P5 Z-stack, the merged view of images with each lectin, *H. somni* strain 2336 EPS, *P. multocida* strain C0513-P5 EPS. **(D)** Polymicrobial biofilm with *P. multocida* C0513 Z-stack, the merged view of images with each lectin, *H. somni* strain 2336 EPS, *P. multocida* strain C0513 EPS.

Fluorescent lectins were used to determine the presence of EPS from each species in the *in vitro* polymicrobial biofilm. Galactomannan EPS from *H. somni* and glycogen EPS from *P. multocida* was detected in the polymicrobial biofilm with FITC-conjugated GS-II and TRITC-conjugated MNA-M, respectively. Glycogen EPS was detected in the polymicrobial biofilm for both *P. multocida* strains tested: biofilm-deficient strain C0513 and biofilm-proficient passed variant C0513-P5 (Figures 3C,D).

COMSTAT analysis of polymicrobial biofilms indicated that 48 h after *P. multocida* C0513 incorporated into an established *H. somni* biofilm, the average thickness of EPS produced by C0513 in the polymicrobial biofilm was 20.73 ± 17.03 μm (Table 2). The average thickness of EPS produced by *H. somni* in the polymicrobial biofilm was 20.58 ± 28.07 μm, indicating variability between the spatial arrangement of EPS within the biofilm. The average biomass and roughness coefficient displayed similar variability. The surface to bio-volume ratios for EPS produced by C0513 and *H. somni* in the polymicrobial biofilm were 5.47 ± 3.16 and 7.21 ± 1.92, respectively. Therefore, although *P. multocida* strain C0513 did not produce substantial biofilm or EPS independently, this strain did produce some EPS and contribution to the polymicrobial biofilm when incorporated into the *H. somni* biofilm.

**TABLE 2:** COMSTAT analysis of fluorescently tagged lectin staining of *in vitro* biofilm EPS.^a^

Polymicrobial	Biomass (μm^3^/μm^2^)	Average Thickness (μm)	Roughness Coefficient (0–2)	Surface to Biovolume ratio (μm^2^/μm^3^)
*P. multocida* C0513	13.46 ± 12.05	20.73 ± 17.03	0.7 ± 0.97	5.47 ± 3.16
*H. somni*	11.2 ± 16	20.58 ± 28.07	1.13 ± 1.03	7.21 ± 1.92
**Polymicrobial**				
*P. multocida* C0513-P5	24.69 ± 11.45	31.98 ± 19.84	0.02 ± 0.01	0.93 ± 0.12
*H. somni*	18.07 ± 10.66	25.06 ± 18.54	0.24 ± 0.62	2.41 ± 2.4
**Single Species**				
*H. somni*	19.69 ± 1.16	26.2 ± 1.99	0.05 ± 0.02	2.98 ± 0.12
*P. multocida* C0513	1.65 ± 0.09	2.31 ± 0.12	0.59 ± 0.04	7.42 ± 0.17
*P. multocida* C0513-P5	19.23 ± 0.68	24.12 ± 0.14	0.04 ± 0.001	2.47 ± 0.25

*^*a*^Data was recorded for biofilms grown 2 days as a single species *H. somni* biofilm, and then for an additional 3 days as a polymicrobial biofilm.*

Polymicrobial biofilms in which passed variant strain C0513-P5 integrated into an established *H. somni* biofilm contained more EPS overall than what was observed for polymicrobial biofilms grown with *P. multocida* strain C0513 (Table 2). COMSTAT analysis 48 h after *P. multocida* strain C0513-P5 was incorporated into the *H. somni* biofilm indicated an average thickness of 31.98 ± 19.84 μm for strain C0513-P5 EPS, and an average thickness of 25.06 ± 18.54 μm for *H. somni* EPS. The average biomass values were also larger when strain C0513-P5 was integrated into the biofilm, and the roughness coefficient was closer to 0 than was observed for polymicrobial biofilms incorporating strain C0513.

Single species biofilms of C0513 and C0513-P5 contained less EPS than what was present in polymicrobial biofilms. The average EPS thickness and biomass for a single species C0513 biofilm was 2.31 ± 0.12 μm and 1.65 ± 0.09 μm^3^/μm^2^, respectively. The average EPS thickness and biomass for a single species C0513-P5 biofilm was 24.12 ± 0.14 μm and 19.23 ± 0.68 μm^3^/μm^2^, respectively (Figure 3A). However, the *H. somni* single species biofilm contained more *H. somni* EPS than polymicrobial biofilms (Figure 3B). The average EPS thickness and biomass for the *H. somni* single species biofilm was 26.2 ± 1.99 μm and 19.69 ± 1.16 μm^3^/μm^2^, respectively.

### Concentrations of Protein and Carbohydrate in Polymicrobial Biofilms

**FIGURE 4:**
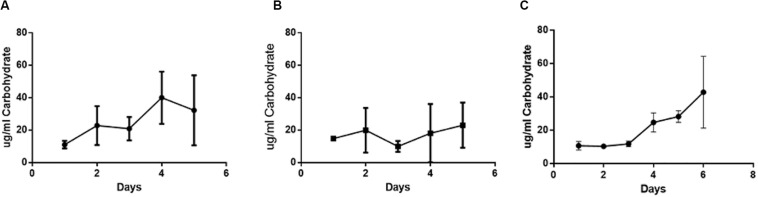
Changes in carbohydrate content during polymicrobial biofilm growth. **(A)** Single species *H. somni* biofilm grown for 5 days. **(B)** Single species *P. multocida* strain C0513-P5 biofilm grown for 5 days. **(C)** Polymicrobial biofilm of *H. somni* grown for 3 days, *P. multocida* added, and growth continued for an additional 3 days. The carbohydrate content of the biofilm was measured by Anthrone assay. All experiments were performed in triplicate in three different experiments (9 datum points total).

Carbohydrate concentrations within the polymicrobial biofilm were determined daily over a period of 6 days (Figure 4). The carbohydrate concentration of the established single species *H. somni* biofilm increased slightly over time, with the greatest increase occurring between days 3 and 4 (Figure 4A). The average carbohydrate concentration of the *P. multocida* single species biofilm remained relatively constant throughout the duration of biofilm growth (Figure 4B). The carbohydrate concentration of the polymicrobial biofilm remained constant until day 3, when *P. multocida* was added to the established *H. somni* biofilm. At this point an increase in the amount of biofilm carbohydrate was measured, and this increase continued until the end of the experiment on day 6 (Figure 4C).

**FIGURE 5:**
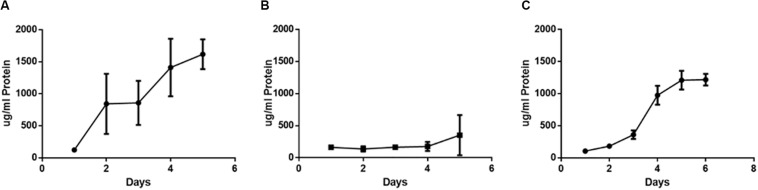
Changes in protein content during polymicrobial biofilm growth. **(A)** Single species *H. somni* strain 2336 biofilm grown for 5 days. **(B)** Single species *P. multocida* strain C0513-P5 biofilm grown for 5 days. **(C)** Polymicrobial biofilm of *H. somni* grown for 3 days, *P. multocida* added, and growth continued for an additional 3 days. The protein content of the biofilms was determined by BCA assay. All experiments were performed in triplicate in three different experiments (9 datum points total).

Protein concentration was measured daily throughout the biofilm growth period (Figure 5). During single species biofilm growth of *H. somni*, protein concentration increased over time until the end of the study at day 5 (Figure 5A). Protein concentration did not increase over time during single species *P. multocida* biofilm growth, and remained at concentrations seen during early stages of biofilm maturation (Figure 5B). Similar to the carbohydrate concentration of the polymicrobial biofilm, the protein concentration of the polymicrobial biofilm increased over time, with the most dramatic increase occurring between days 3 and 4, which coincided with the addition of *P. multocida* to the established *H. somni* biofilm on day 3 (Figure 5C).

### Auto-Aggregation of Polymicrobial Cultures

**FIGURE 6:**
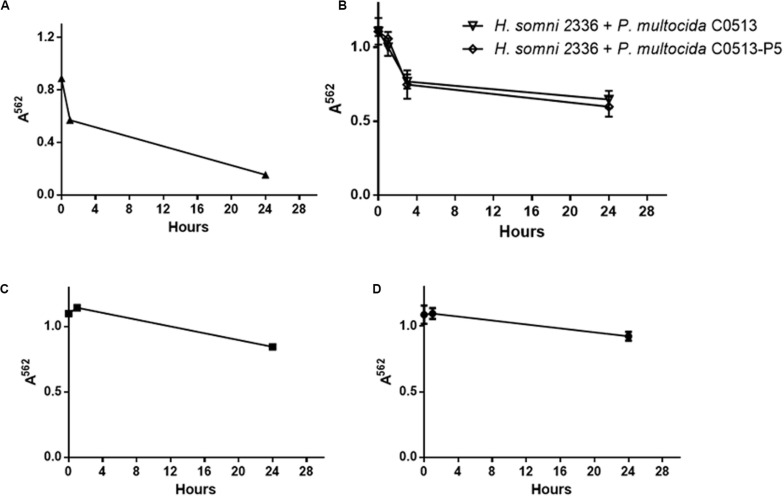
Auto-aggregation of polymicrobial suspensions over 24 h. **(A)** Auto-aggregation of *H. somni* strain 2336 as a single species. **(B)** Auto-aggregation between *H. somni* strain 2336 and *P. multocida* strain C0513 or strain C0513-P5. **(C)** Auto-aggregation of *P. multocida* strain C0513-P5. **(D)** Auto-aggregation of *P. multocida* strain C0513. All experiments were performed in triplicate in three different experiments (9 datum points total).

Adhesion is the first stage of biofilm formation, and auto-aggregation can be used as a measure of cellular adhesion (Felek et al., 2008; Sorroche et al., 2012; Arenas et al., 2015). In this study, auto-aggregation of polymicrobial bacterial suspensions was used to determine if interactions between the two species occurred. *H. somni* auto-aggregated 80% within 24 h in a single species suspension (Figure 6A), while *P. multocida* strains C0513-P5 and C0513 auto-aggregated significantly less: 29 and 18% within 24 h (Figures 6C,D, respectively), and was confirmed by viable plate count (data not shown). No additional auto-aggregation was observed for *P. multocida* or *H. somni* after 24 h incubation (data not shown). Polymicrobial bacterial suspensions auto-aggregated 50% when either the encapsulated (strain C0513) or capsule-deficient strain *P. multocida* (C0513-P5) was used (Figure 6B), suggesting that interaction occurred between *H. somni* and *P. multocida* when in a polymicrobial environment. Polymicrobial auto-aggregation experiments carried out in the presence of crudely extracted *H. somni* biofilm material did not display aggregation (data not shown), indicating that cells must be present to observe this effect.

### Polymicrobial Biofilm Formation *in vivo*

**FIGURE 7:**
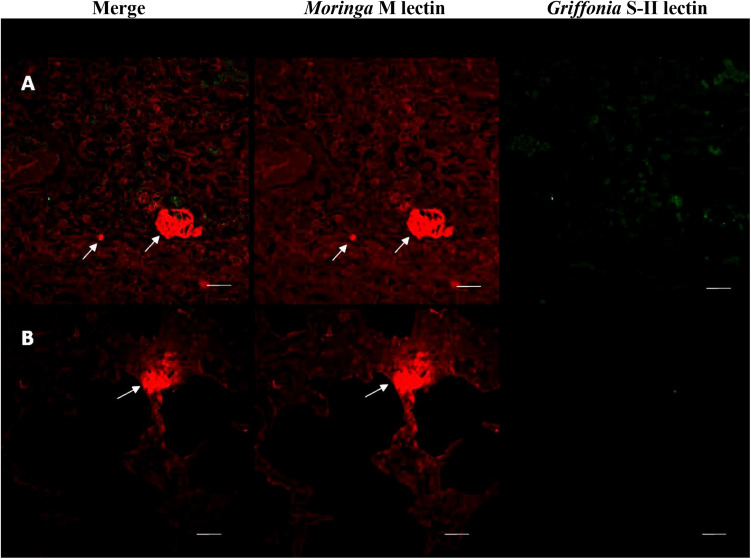
Fluorescently tagged lectin staining of *in vivo* biofilm EPS. Lung tissue sections from calf 65 stained with fluorescently tagged lectins. **(A,B)** Two separate samples showing merged images of images using each lectin, section stained with *Moringa* M lectin labeled with TRITC for EPS from *H. somni* only, section stained with *Griffonia simplicifolia* labeled with FITC for EPS from *P. multocida* only. The *H. somni* EPS is only made during biofilm formation indicating the presence of a biofilm in these sections. Scale bar is equal to 20 um.

Lung tissue from calves experimentally infected with *H. somni* was analyzed for polymicrobial biofilm formation by histopathology. Previous experimental infections have shown that *P. multocida* can be isolated from the lower respiratory tract of calves in conjunction with *H. somni* following experimental challenge with *H. somni* alone (Gogolewski et al., 1987a, b; Elswaifi et al., 2012; Murray et al., 2017). Both *H. somni* and *P. multocida* were isolated from calf 65, but not calf 66. However, PCR revealed *P. multocida* and *H. somni* DNA present in the lungs of both calves 65 and 66. *H. somni* and *P. multocida* could not be detected by F.I.S.H. *in vivo*. However, fluorescent lectin staining did demonstrate the presence of biofilm matrix-specific EPS produced by *H. somni* in calf 65, but not *P. multocida* EPS (Figure 7).

### Histological Analysis of Polymicrobial Respiratory Disease

Tissue sections from calf 65 and 66 were observed for histological analysis. In both calves the lungs appeared slightly inflamed and partially collapsed. Sections of lung contained pleural fibrosis. Neutrophils and macrophages were seen in lung airways, and lymphocytes were present at the lung periphery. Acute pulmonary edema was present in the interlobular septa, and bronchioles displayed minor hemorrhaging (data not shown).

### Enzyme-Linked Immunosorbent Assay

Calves 65 and 66 produced an antibody titer to *H. somni* planktonic cells of 1:20. However, both calves produced an antibody titer of 1:800 to the *H. somni* biofilm. Both calves produced an antibody titer to *P. multocida* planktonic cells of 1:1600, and a titer of 1:3200 to the *P. multocida* biofilm. There was no antibody titer to *P. multocida* or *H. somni* detected prior to challenge with *H. somni*.

### RNA-Seq Analysis

Previous RNA-Seq analysis of *H. somni* indicated that up to half of the bacterial genes are significantly differentially expressed when the bacteria are grown in a biofilm, compared to planktonic growth (Petruzzi et al., 2015). Such differential regulation was also observed with *P. multocida* when grown as a polymicrobial biofilm compared to a single species biofilm. A total of 282 genes were differentially regulated when *P. multocida* was grown as a biofilm together with *H. somni* compared to growth as a mono-species biofilm. Examples of some genes that were significantly upregulated are shown in Table 3. Many of these genes encode for proteins involved in iron acquisition or outer membrane composition, including lipopolysaccharide (LPS).

**TABLE 3:** Examples of genes significantly differentially regulated when *P. multocida* was grown in a poly-microbial biofilm with *H. somni* compared to a monophasic biofilm.^a^

Gene function	Fold increase	Product	Locus tag
**Iron acquisition**			
*exbB*	3.78	Ferric siderophore transport system biopolymer transport protein	P1059-01334
*exbD*	3.80	Biopolymer transport protein	P1059-01135
*iscA*	3.48	Iron-sulfur assembly iron binding protein	P1059-00347
	2.41	Ferric iron ABC transporter permease protein	P1059-00050
*fur*	1.31	Ferric uptake regulation protein	P1059-00379
*plpB*	1.30	Lipoprotein B	P1059-01782
**Inner membrane**			
*tolA*	9.75	TolA protein	P1059-01107
**Outer membrane**			
*bamC*	5.32	Outer membrane beta-barrel assembly protein	P1059-01195
*bamD*	7.56	Outer membrane beta-barrel assembly protein	P1059-01888
*bamE*	6.53	Outer membrane beta-barrel assembly protein	P1059-02059
	2.80	Outer membrane lipoprotein	P1059-00601
**Enzymes that synthesize lipopolysaccharide some proteins**			
	2.58	Outer membrane stress serine protease	P1059-00859
	2.06	3-deoxy-manno-octulosonate cytidylyltransferase	P1059-00989
	6.28	UDP-N-acetylglucosamine 1-carboxyvinyltransferase	P1059-00194
	3.93	Acyl-[acyl-carrier-protein]–UDP-N-acetylglucosamine O-acyltransferase	P1059-02153
	3.81	2-Keto-3-deoxy-D-manno-octulosonate-8-phosphate synthase	P1059-00605
	6.48	Lipid-A-disaccharide synthase	P1059-02154
*rpoH*	3.46	RNA polymerase sigma factor	P1059-01754
	2.65	Sigma factor RpoE negative regulatory protein RseB precursor	P1059-01958
*ibaG*	5.49	Acid stress protein IbaG	P1059-00193

*^*a*^In total, 282 genes were significantly differentially regulated in *P. multocida* when grown as a polymicrobial biofilm with *H. somni* compared to growth as a monophasic biofilm.*

The expression of host genes at the infected site were compared to the same genes at normal tissue sites of the same lung. Ninety-three genes were expressed greater than 2.5-fold at the infected site, most of which are involved in inflammation and the host immune response. A partial list of the gene ontology (GO) terms and these genes is listed in Table 4. Genes that were upregulated most significantly were those encoding for cytokines and chemokines that contribute to inflammation and the host immune response, chemotaxis and differentiation of phagocytic cells, and other related cellular catabolic processes.

**TABLE 4:** Gene Ontology (GO) terms enriched among 93 genes expressed >2.5 fold higher in infected site.^a^

GO term	Genes	*P*-value	FDR-adj
Collagen catabolic process	MMP9, CTSB, MMP14, MMP13, MMP1	0.0001	0.0002
Inflammatory response	SLC11A1, IL17A, CCL20, CXCL5, CCR1, TLR2, NOS2,		
	CD14, S100A12	0.0001	0.0071
Innate immune response	S100A8, BPIFA1, S100A9, TLR2, SLPI, CD14, S100A12	0.0020	0.2485
Cell chemotaxis	CCL20, CXCL5, SAA3, M-SAA3.2	0.0025	0.2361
Proteolysis	CNDP2, MMP9, LGMN, MMP13, MMP1, PLAU	0.0034	0.2545
Immune response	CSF3, CXCL5, ENPP3, CCR1, IL1B, FTH1	0.0081	0.4405
Endodermal cell differentiation	INHBA, MMP9, MMP14	0.0098	0.4524
Cellular response to triacyl bacterial lipopeptide	TLR2, CD14	0.0115	0.4618
Neutrophil aggregation	S100A8, S100A9	0.0115	0.4618
Positive regulation of peptide secretion	S100A8, S100A9	0.0115	0.4618
Cellular iron ion homeostasis	SLC11A1, TFRC, FTH1	0.0155	0.5245
Proteolysis involved in cellular protein catabolic process	CTSK, LGMN, CTSB	0.0174	0.5272
Peptidyl-cysteine S-nitrosylation	S100A8, NOS2	0.0230	0.5939
Cellular response to diacyl bacterial lipopeptide	TLR2, CD14	0.0230	0.5939
Positive regulation of inflammatory response	S100A8, S100A9, TLR2	0.0245	0.5866
Neutrophil chemotaxis	CCL20, S100A8, S100A9	0.0302	0.6351
Cellular response to zinc ion	MT1A, MT2A	0.0342	0.6543
Cellular response to lipoteichoic acid	TLR2, CD14	0.0342	0.6543
Epithelial cell differentiation	KRT5, KRT14, CTSB	0.0377	0.6649
Leukocyte migration involved in inflammatory response	S100A8, S100A9	0.0398	0.6619
Nitric oxide mediated signal transduction	MT2A, NOS2	0.0454	0.6886

*^*a*^This is a partial list of 93 genes that were expressed >2.5-fold at the infected site, compared to healthy sites within the same lung. Other GO terms of genes enriched, but <2.5-fold, are not listed.*

## Discussion

Despite available vaccines, BRD is still the leading cause of morbidity and mortality in the North American beef and dairy industries. Research over the past 40 years has aided our understanding of the transmission and pathogenesis of the causative agents of BRD, but the disease and improved vaccines and treatments remain in need (Griffin, 1997; Stovall et al., 2000). BRD is a multifactorial disease, and diagnosis is often complicated by the presence of several potential etiologic agents, many of whom are opportunistic pathogens. The 4 most common bacterial agents of BRD (*H. somni, P. multocida, M. haemolytica*, and *M. bovis*) have been shown to be capable of forming biofilms (Sandal et al., 2007; Simmons and Dybvig, 2009; Boukahil and Czuprynski, 2015; Petruzzi et al., 2017). Some species, such as *H. somni*, have been documented to also form biofilms in cardiopulmonary tissue during BRD (Sandal et al., 2009; O’Toole and Sondgeroth, 2016; O’Toole et al., 2017). In order to further understand the potential role of biofilms in BRD, we have characterized the polymicrobial relationship between two of these important BRD agents: *H. somni* and *P. multocida*.

The adherence of bacteria to a framework or scaffolding, such as a biofilm matrix that is assembled by different bacterial species or by the host, is known as coadhesion (Bos et al., 1994; Rickard et al., 2003). Preliminary studies in our lab indicated that the ideal conditions for polymicrobial biofilm formation between *H. somni* and *P. multocida* were created by allowing *H. somni* to establish a preliminary biofilm before the addition of the secondary colonizer, *P. multocida*. This is consistent with experimental results obtained with other bacterial agents that formed coadhesive polymicrobial biofilms (Bos et al., 1994; Rickard et al., 2003; Castonguay et al., 2006; Karched et al., 2015). *P. multocida* was not inhibitory to *H. somni*; but grew much faster and if growth of the two species was initiated at the same time, *P. multocida* simply over-grew *H. somni*.

Using labeled DNA probes the spatial arrangement of the bacterial species within the polymicrobial and single species biofilms could be observed, and allowed the monitoring of bacterial growth over time. Antibodies raised against *H. somni* or *P. multocida* surface antigens displayed cross reactivity, limiting the microscopic and fluorescent techniques available for discernable observation (Pan et al., 2014). The use of specific DNA probes allowed for the distinct identification of DNA (and hence the cells) from each species simultaneously (Thurnheer et al., 2004). Although we cannot negate the possibility that 16S rRNA DNA was present in the extracellular matrix, we presume that the DNA probes detected predominately, or only cellular-associated, DNA (Okshevsky and Meyer, 2015). Over a 48-h period of polymicrobial growth, the DNA present from both species increased and did not appear to be separated into large micro-colonies or separate biofilms, indicating an integrated polymicrobial biofilm had formed.

The polymicrobial relationship of the two species was further confirmed using fluorescently tagged lectins specific for the EPS produced by *H. somni* or *P. multocida.* Interestingly, the use of lectins provided insight into the glycogen synthesis of a *P. multocida* capsule-producing bovine-associated strain: C0513. This strain did not produce a substantial single-species biofilm. However, once incorporated into the *H. somni* biofilm matrix *in vitro*, it began producing EPS and contributing to the overall biofilm matrix. These results indicate that encapsulated *P. multocida* that cannot efficiently produce a biofilm on its own can contribute to biofilm formation in the *H. somni* biofilm, which may be advantageous to both species. The capsule-deficient passed variant of this strain, C0513-P5 did produce a single species biofilm, as previously reported (Petruzzi et al., 2017), and produced visibly more EPS when incorporated into the *H. somni* biofilm matrix than in a single species biofilm. However, the amount of EPS produced by *H. somni* in the polymicrobial biofilm, compared to when grown as a single species, decreased. The reason for this was not determined, but may be due to a synergistic relationship with *P. multocida*, resulting in a decreased need for *H. somni* EPS (Castonguay et al., 2006).

The overall biofilm matrix carbohydrate content was determined to increase over time, further supporting that the incorporation of *P. multocida* into the *H. somni* biofilm had an additive effect on the matrix. However, there was little protein content in the single species *P. multocida* biofilm, and no additive protein to the polymicrobial biofilm, suggesting that, unlike most biofilms, protein may not be a major component of the *P. multocida* biofilm. This observation needs to be confirmed. While the majority of *H. somni* cells auto-aggregated within 24 h, only the minority of *P. multocida* cells did. Encapsulated cells auto-aggregated less than capsule-deficient cells, indicating that the capsule (likely the electrostatic negative charge of the capsule) interfered with auto-aggregation. We presume that although capsule-deficient, strain C0513-P5 also had a substantially more negative surface charge than *H. somni.* Polymicrobial aggregation experiments to determine interactions between *H. somni* and *P. multocida* cell surfaces showed that aggregation of the two species together was greater than the aggregation of *P. multocida* alone, suggesting that *H. somni* interacted with *P. multocida* to enhance the aggregation of *P. multocida.* However, there was no effect if only *H. somni* biofilm material was added, indicating cell-cell interaction was occurring. Further experimentation on species interactions is warranted.

Calves challenged with *H. somni* formed a polymicrobial respiratory infection with both *H. somni* and *P. multocida*, even though the calves were raised in isolation from the day of birth and challenged with only *H. somni.* This polymicrobial interaction has been reported in previous experimental infections (Gogolewski et al., 1987a, b; Elswaifi et al., 2012; Murray et al., 2017). Although the calves presented with only mild symptoms of respiratory disease, at necropsy histological analysis revealed that both calves had a respiratory infection consistent with experimental BRD. The lungs appeared inflamed and slightly collapsed, and contained pleural fibrosis. However, lesions were only visible in one lobe of both calves.

Lung sections taken from visible lesions were microscopically observed by the same techniques used for *in vitro* polymicrobial biofilms. DNA from neither *H. somni* nor *P. multocida* were detected in the lungs of calves using F.I.S.H., but DNA from both species was detected in the lungs of calves by PCR. *H. somni* biofilm EPS was detected in the lungs of one calf, and EPS is produced by *H. somni* only during biofilm formation (Sandal et al., 2007). However, EPS produced by *P. multocida* was not detected in the lungs of either calf. *P. multocida* EPS may have been masked due to the presence of capsular polysaccharide, which is required for full virulence *in vivo*. However, both calves produced an antibody response to crude *P. multocida* biofilm material, indicating the calves were exposed to biofilm matrix material produced by *P. multocida* during infection. These results support the *in vitro* results that showed encapsulated *P. multocida* could generate a biofilm when in the *H. somni* biofilm. Additionally, calves produced a weaker antibody response to *P. multocida* whole planktonic cells, a much weaker response to *H. somni* whole planktonic cells, and the most robust response to the *P. multocida* and *H. somni* biofilm matrixes. These results support that both *P. multocida* and *H. somni* were present predominately as a biofilm during chronic BRD infection.

Bacterial gene expression is highly regulated and heavily influenced by environmental factors (Balleza et al., 2009). As described previously and reported here, not only does biofilm formation alter gene expression, in comparison to planktonic growth, but growth in a polymicrobial biofilm further alters gene expression, in comparison to a mono-species biofilm. Likely as a result of competition for resources it is not surprising that many genes, such as those involved in regulation and iron acquisition, were significantly upregulated when *P. multocida* was grown in a biofilm with *H. somni*. Furthermore, outer membrane proteins play a major role in transport of molecules across the outer membrane (Koebnik et al., 2000), and the LPS plays an important role in outer membrane permeability and integrity (Bertani and Ruiz, 2018). Therefore, while not inhibiting *H. somni*, it appeared that *P. multocida* altered gene expression to obtain a competitive advantage, and to persist and contribute to the developing *H. somni* biofilm.

The host response at the site of infection, compared to healthy tissue, was enhanced for the expression of genes associated with inflammation and the immune response, including multiple cytokines and chemokines. Of interest was that while there was an enhanced response by TLR2, there was not a measured differential response by TLR4, indicating the response to bacterial proteins was greater than to LPS. However, because we could not control the polymicrobial nature of the biofilm *in vivo* we could not determine if the host response to the polymicrobial infection was different than it would have been to a mono-species infection. Biofilms associated with infections of animals are a significant problem that have been poorly studied (Clutterbuck et al., 2007). Reports of polymicrobial biofilms of veterinary importance are even less common. However, the relevance of polymicrobial biofilms to human medicine has become well established (Brogden et al., 2005; Peters et al., 2012). This is the first report of a coadhesive polymicrobial biofilm relationship associated with BRD, leading us to suspect polymicrobial biofilm interactions between other bacterial agents of BRD may also occur. Polymicrobial BRD has been reported, but the presence of biofilm was not considered (Booker et al., 2008; Hotchkiss et al., 2010; Holman et al., 2017; Murray et al., 2017). While further research to reduce losses caused by BRD has been recommended (Griffin, 1997; Fulton, 2009; Taylor et al., 2010), much of this new work should be focused on means to treat or prevent biofilm infections.

## Data Availability Statement

The databases presented in this study can be found in the NCBI SRA online repository https://www.ncbi.nlm.nih.gov/sra/?term=SRR11948555.

## Ethics Statement

The animal study was reviewed and approved by the Virginia Tech Institutional Animal Care and Use Committee (Approval number 16-128 CVM).

## Author Contributions

BP carried out most of the experimental work and wrote and edited the manuscript. KL harvested the tissues and performed gross and histopathologic pathology of the calves. AD carried out bioinformatic analyses of the RNA-Seq results, and contributed data to the manuscript. WS challenged, monitored, and collected samples from the calves. TI designed the experiments, contributed to writing the manuscript, and did the final editing of the manuscript. All authors contributed to the article and approved the submitted version.

## Conflict of Interest

The authors declare that the research was conducted in the absence of any commercial or financial relationships that could be construed as a potential conflict of interest.

## Footnotes

1 https://david.ncifcrf.gov/

2 https://www.ncbi.nlm.nih.gov/sra/?term=SRR11948555
